# Efficacy and safety of selective glucocorticoid receptor modulators in comparison to glucocorticoids in arthritis, a systematic review

**DOI:** 10.1371/journal.pone.0188810

**Published:** 2017-12-21

**Authors:** M. Safy, M. J. H. de Hair, J. W. G. Jacobs, F. Buttgereit, M. C. Kraan, J. M. van Laar

**Affiliations:** 1 Department of Rheumatology & Clinical Immunology, University Medical Center Utrecht, Utrecht, the Netherlands; 2 Department of Rheumatology and Clinical Immunology, Charité—Universitätsmedizin Berlin, Berlin, Germany; 3 Department of Rheumatology and Inflammation Research at Institute of Medicine, University of Gothenburg, Gothenburg, Sweden; Northwestern University, UNITED STATES

## Abstract

**Background:**

Long-term treatment with glucocorticoids (GCs) plays an important role in the management of arthritis patients, although the efficacy/safety balance is unfavorable. Alternatives with less (severe) adverse effects but with good efficacy are needed. Selective GC receptor modulators (SGRMs) are designed to engage the GC receptor with dissociative characteristics: transactivation of genes, which is mainly responsible for unwanted effects, is less strong while trans-repression of genes, reducing inflammation, is maintained. It is expected that SGRMs thus have a better efficacy/safety balance than GCs. A systematic review providing an overview of the evidence in arthritis is lacking.

**Objective:**

To systematically review the current literature on efficacy and safety of oral SGRMs in comparison to GCs in arthritis.

**Methods:**

A search was performed in Medline, Embase and the Cochrane Library, from inception dates of databases until May 2017. Experimental studies involving animal arthritis models or human material of arthritis patients, as well as clinical studies in arthritis patients were included, provided they reported original data. All types of arthritis were included. Data was extracted on the SGRM studied and on the GC used as reference standard; the design or setting of the study was extracted as well as the efficacy and safety results.

**Results:**

A total of 207 articles was retrieved of which 17 articles were eligible for our analysis. Two studies concerned randomized controlled trials (RCT), five studies were pre-clinical studies using human material, and 10 studies involved pre-clinical animal models (acute and/or chronic arthritis induced in mice or rats). PF-04171327, the only compound investigated in a clinical trial setting, had a better efficacy/safety balance compared to GCs: better clinical anti-inflammatory efficacy and similar safety.

**Conclusion:**

Studies assessing both efficacy and safety of SGRMs are scarce. There is limited evidence for dissociation of anti-inflammatory and metabolic effects of the SGRMs studied. Development of many SGRMs is haltered in a preclinical phase. One SGRM showed a better clinical efficacy/safety balance.

## Introduction

Glucocorticoids (GCs) are the most commonly used anti-inflammatory drugs worldwide, applied in arthritic diseases, inflammatory bowel disease, and chronic pulmonary disease, for example [1–3]. In rheumatoid arthritis (RA) between 56% and 68% of the patients are treated with GCs [4–6]. GCs not only exhibit anti-inflammatory effects, but also have proven disease modifying effects as they halter radiological damage and improve physical disability in RA patients in addition to reducing disease activity [7–9]. Despite their proven beneficial effects, GCs potentially cause adverse effects. The most common adverse effects associated with GC use are cardiovascular events, endocrine/metabolic effects (weight gain, dysregulation of glucose metabolism and development of diabetes), infections, gastro-intestinal events and osteoporosis [10–11]. These unwanted effects especially occur when used long-term (>6 months) and in high-dose (>10 mg/daily),and limit the dosing and duration of GC treatment [12]. Hence, the quest for alternatives with a better efficacy/safety balance continues, such as selective GC receptor modulators (SGRMs). SGRMs are specifically designed to engage the GC receptor (GR) with dissociative characteristics: after binding to the GR, GCs may either bind to and activate transcription from gene promoters (transactivation) or interact with other transcription factors to change their function (transrepression). It is assumed that SGRMs promote transrepression over transactivation [13].

Transrepression is most critical for the anti-inflammatory effects of GCs, as it leads to decreased production of pro-inflammatory transcription factors such as nuclear factor-kappa B (NF-κB) and activator protein 1 (AP-1). On the contrary, transactivation is thought to cause detrimental effects of GCs [14]. Upon binding of a GC to GC response elements (GRE) transactivation in various gene promotors occurs, such as glucose-6-phosphatase (G6Pase), phosphoenolpyruvate carboxykinase (PEPCK), fatty acid synthase (FAS) and tyrosine aminotransferase (TAT). The protein products of these genes are involved in carbohydrate, lipid and protein metabolism [15]. As such, activation of these genes could lead to aforementioned (metabolic) side effects.

SGRMs may have an improved efficacy/safety balance compared to conventional GCs, by their potentially disparate effects on transrepression and transactivation. However, till date, no SGRM has entered the market yet, suggesting that development of SGRMs meets challenges. A systematic review providing an in-depth overview of both the efficacy and safety of SGRMs is lacking. Our aim was therefore to systematically investigate whether oral SGRMs have a superior efficacy/safety balance compared to conventional GCs in arthritis in (pre)clinical settings.

## Methods

### Search and selection

A systematic literature search was performed, to assess efficacy and safety of oral SGRMs in arthritis, compared to GCs. MEDLINE (PubMed), Embase and the Cochrane Library were searched until May 2017. The search (S1 Box) was established after consultation of a librarian at the University Medical Center (UMC) Utrecht with expertise in systematic literature searches (P.H.W.*)*. Duplicates were excluded. Two authors (M.S. and M.J.H.H.) independently screened titles and abstracts for eligibility. Studies were included if fulfilling the following criteria: investigating efficacy and safety of an oral SGRM; studying GC as reference compound; performed in arthritis. Both in vivo and in vitro, were included. Subsequently, the same authors independently screened full texts of eligible articles. Selection was based on mutual agreement. Studies were excluded if not investigating a GC as reference compound; if performed in non-arthritic disease(s); if investigating non-selective GRMs, or if investigating SGRMs with administration route other than oral. Review articles without presentation of original data were also excluded. Of the selected articles, references and citing publications were additionally screened.

### Data extraction

Data was extracted using the SYstematic Review Centre for Laboratory animal Experimentation (SYRCLE’s) guideline ([16]. This guideline is adapted from the Cochrane risk of bias tool [17] and focuses on laboratory animal studies. We extracted data on the SGRM investigated (experimental compound), the GC that was investigated as reference compound, the animal model or setting of the study, and efficacy and safety results. Initial data extraction was performed by one author (M.S.) and extracted data was re-assed by the second author (M.J.H.H.). The efficacy results concerned pro- and anti-inflammatory effects and the safety results concerned any adverse effect reported, including effects on glucose, fat and bone metabolism, as well as mineralocorticoid effects. For clinical studies, also results on adverse effects of GC were extracted, such as cardiovascular events and infections. Results were reported following the Preferred Reporting Items for Systematic Reviews and Meta-analysis (PRISMA) checklist [18].

## Results

### Search and selection

**Fig 1:**
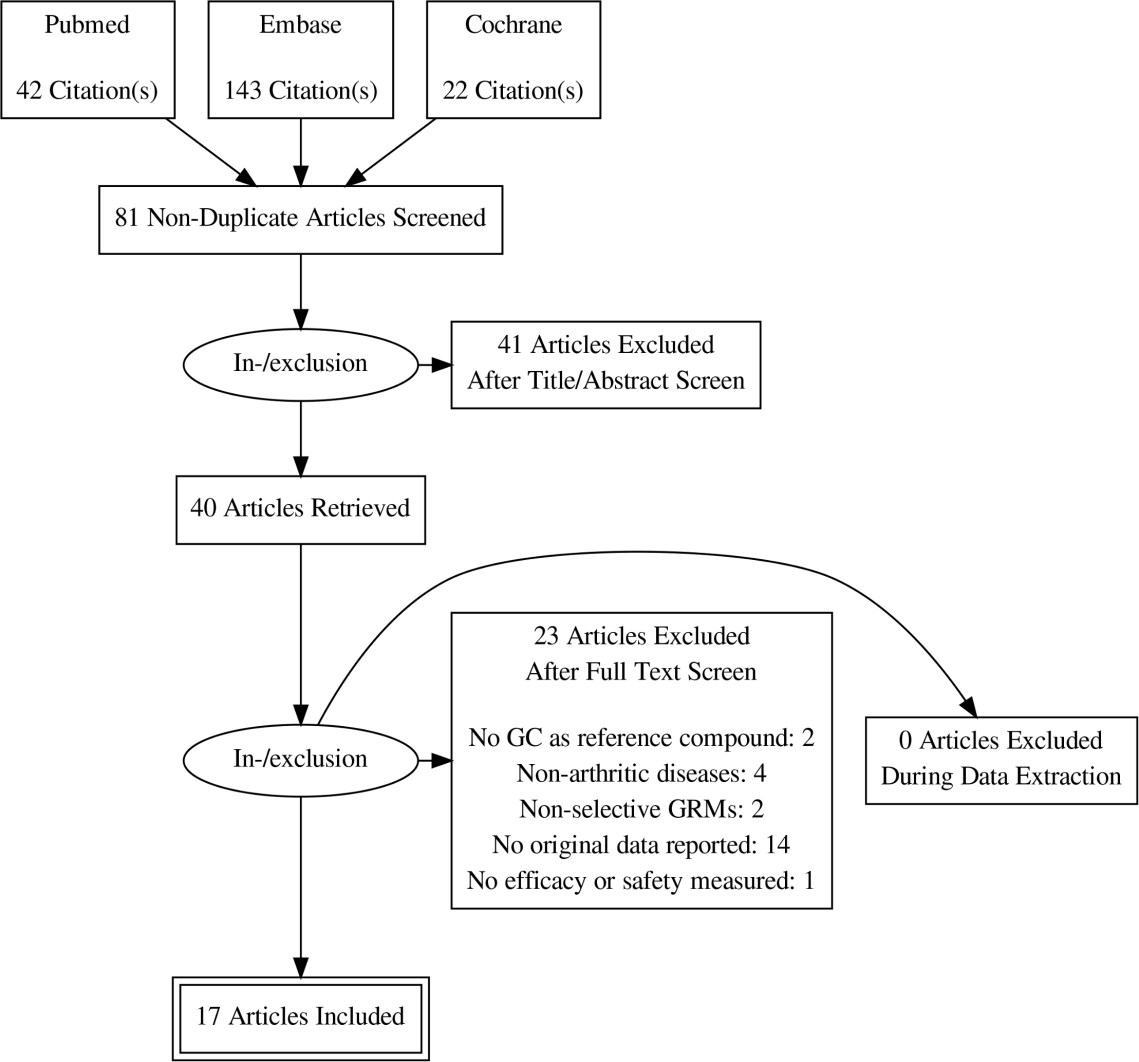
Flow chart of search and selection of studies on efficacy and safety of SGRMs. SGRMs: selective GRMs; GRMs: glucocorticoid receptor modulators; GCs: glucocorticoids.

The search and selection is presented in Fig 1. A total of 207 reports was retrieved by the initial search. Excluding duplicates and reports other than articles resulted in 81 articles, of which title and abstract were screened. This resulted in 40 articles of which full text was screened. Finally, 17 articles were eligible for inclusion and analysis.

Risk of bias was not assessed, because of very high heterogeneity in study types and in study design and information presented.

### Data extraction

Study characteristics are shown in Table 1. Results are reported using the PRISMA checklist (S1 Table). The following SGRMs were investigated: Compound A, PF-04171327, LGD-5552, Compounds 4, 5, and 14, Compounds (R)-16, (R)-18, (R)-21, (R)-35, and (R)-37, Ginsenoside Rg1 and Org 214007–0 [19–35].

**Table 1:** Overview of studies with efficacy or safety results of a selective glucocorticoid receptor modulator in comparison to a glucocorticoid.

Author, year	SGRM tested	Reference GC	Setting	Safety		Conclusion on efficacy and safety in comparison to a glucocorticoid
				Glucose homeostasis/ fat metabolism	Bone markers	
**Dewint et al., 2008**	Compound A	Dex	B, C	**X**		Similar efficacy. Better safety of SGRM.
**Gossye et al., 2009**	Compound A	Dex	B			Similar efficacy. No safety data shown.
**Gossye et al., 2010**	Compound A	Dex	B, C			Lower efficacy of SGRM. No safety data shown.
**Rauch et al.,2011**	Compound A	Dex	B		**X**	Similar efficacy. Better safety of SGRM.
**Rauner et al., 2013**	Compound A	Dex	C		**X**	Lower efficacy of SGRM. Better safety of SGRM.
**Malaise et al., 2015**	Compound A	Pred	B	**X**		Similar efficacy. Better safety of SGRM.
**Yang et al.,2015**	Compound 4 and 5	Pred	C			Similar efficacy of compound 4, better efficacy of compound 5. No safety data shown.
**Razavi et al., 2014**	Compound 14	Pred	C	**X**		Similar efficacy. Better safety of SGRM.
**Riether et al., 2010**	Compounds (R)-16 and (R)-37	Pred	C	**X**		Similar efficacy. Better safety of SGRM.
**Harcken et al., 2014**	Compounds (R)-18 and (R)-21	Pred	C	**X**	**X**	Similar efficacy. Better safety of SGRMs.
**Weinstein et al., 2011**	Compound35 and 37	Pred, Dex	C			Better efficacy of SGRMs. No safety data shown.
**Miner et al., 2007**	LGD-5552	Pred	C			Similar efficacy. No safety data shown.
**Lopez et al.,2008**	LGD-5552	Pred	C			Similar efficacy. No safety data shown.
**Du et al., 2011**	Ginsenoside Rg1	Dex	C	**X**	**X**	Similar efficacy. Better safety of SGRM.
**Van Lierop et al., 2012**	Org 214007–0	Pred	C			Similar efficacy. No safety data shown.
**Conrado et al., 2015**	PF-04171327	Pred	D			Similar efficacy. No safety data shown.
**Stock et al., 2017**	PF-04171327	Pred	A	**X**	**X**	Better efficacy of SGRM. Similar safety.

Studies are sorted on type of SGRM. Efficacy was measured in all 17 studies, safety was measured in nine studies. SGRM: selective glucocorticoid receptor modulator; GC: glucocorticoid; Pred: prednisone; Dex: dexamethasone; A: randomized controlled trial (RCT); B: pre-clinical study with human material; C: pre-clinical study with animal material/model; D: stochastic simulations based on non-published RCT

Of the 17 studies, 2 studies concerned randomized controlled trials (RCT), 3 were pre-clinical studies using only human material, 10 studies were performed using only a pre-clinical animal model (acute and/or chronic arthritis induced mice or rat model) and 2 pre-clinical studies used both animal as well as human material. Regarding the animal models, an acute arthritis induced model was used to measure pro-inflammatory cytokines, and a chronic induced arthritis model to measure a clinical outcome, such as paw swelling. Dexamethasone was used as reference GC in 7 studies, and prednisone in 11 studies.

Seven studies showed better safety of the studied SGRM compared to dexamethasone or prednisone, with similar efficacy. Four compounds showed similar efficacy of the studied SGRM compared to prednisone, but no safety data was provided. Three compounds showed better efficacy of the studies SGRM than prednisone or dexamethasone, but no safety data was provided for two compounds. In depth results of 6 studies that assessed both efficacy and safety of SGRMs in comparison to GCs are depicted in Table 2 and only studies reporting efficacy and safety results of both SGRMs and GCs were included in this table. Fosdagrocorat (PF-04171327), was the only compound investigated in a clinical setting [35], and of this SGRM both safety and efficacy data was available. In this phase 2 study, 86 RA patients were randomized to receiving either 10 mg or 25 mg fosdagrocorat, or 5 mg prednisone or placebo. A significantly better improvement in DAS28-CRP was observed after two weeks of treatment with 25 mg fosdagrocorat compared to 5 mg prednisone and placebo. Treatment with the 10 mg dose of fosdagrocorat was only compared to placebo, not prednisone. Plasma cortisol levels decreased significantly more in the group of patients treated with 10 mg and 25 mg fosdagrocorat compared to 5 mg prednisone. The number of adverse events was similar between the group of patients receiving 25 mg fosdagrocorat compared to 5 mg prednisone.

**Table 2:** Details of studies with data on both efficacy and safety of selective glucocorticoid receptor modulator in comparison to a glucocorticoid.

Author, year	SGRM tested	GC	Setting/model	Efficacy (SGRM compared to GC)		Safety (SGRM compared to GC)	
**Dewint et al., 2008**	Compound A	Dex	FLS cells derived from RA patients	Amount of cDNA of TNF =	NA		
				Amount of cDNA of MMP1 =	NA		
				Amount of cDNA of MMP3 =	NA		
			CIA mice	Arthritis score at day 8 of arthritis ↑	NA	Serum levels of insulin ↓	NA
				Paw swelling at day 8 of arthritis =	NA	Normal histology of knee joints ↓	NA
						mRNA G6P ↓	NA
						mRNA PEPCK ↓	NA
**Rauner et al., 2013**	Compound A	Dex	CIA mice	Arthritis score ↑	NA	Bone loss ↓	NA
				Paw swelling ↑	NA	Serum P1NP ↓	NA
				Paw temperature ↓	NA	Serum CTX-1 ↓	NA
						Cellular infiltration in paws ↓	NA
						Cartilage destruction ↓	NA
						Inhibition of number of osteoclasts ↓	NA
			Supernatant from PBMCs from mice, ex vivo stimulated with collagen type II	TNF =	NA		
				IFN-a =	NA		
				NF-ƙβ =	NA		
			mRNA expression in joint tissue from CIA mice	TNF ↑	NA		
				IL-6 ↓	NA		
**Razavi et al., 2014**	Compound 14	Pred	Mice inflammation model, LPS stimulated	IL-6 ↓	NA		
				TNF =	NA		
			CIA mice	Arthritis score ↑	NA	Insulin ↑	NA
						Body fat ↑	NA
						Triglycerides ↓	NA
						Free fatty acids ↑	NA
**Harcken et al., 2014**	Compound R18 and 21	Pred	CIA mice	Arthritis score ↓	NA	Osteocalcin ↑	NA
						Body fat ↑	NA
						Triglycerides ↑	NA
						Free fatty acids ↑	NA
						Insulin ↑	NA
						Femur cortical thickness =	S
**Du et al., 2011**	Ginsenoside Rg1	Dex	Inflamed paw model	Paw swelling ↑	NS		
			CIA mice	Arthritis score =	NA	Body weight ↓	NA
						Blood glucose levels ↓	S
						Bone cortical thickness ↑	NA
						Bone content ↑	S
						Trabecular tibial number ↑	S
						Trabecular tibial thickness ↑	NS
						Trabecular tibial separation ↑	S
**Stock et al., 2017**	PF-04171327	Pred	Phase 2 RCT, 86 RA patients, 2 weeks treatment	DAS28-4 (CRP) improvement ↑	S	Fasting glucose =	NS
						Plasma cortisol ↑	S
						Adverse events =	NA
						Mean osteocalcin levels =	NA
						Mean uNTX-1 levels =	NA

Six studies reporting results on both efficacy and safety of SGRMs compared to GCs are depicted, only studies that reported efficacy and safety of both SGRM and GC are shown in this table. Results have been summarized for each SGRM if multiple dosing schemes were used. SGRM: selective glucocorticoid receptor modulator; GC: glucocorticoid; pred: prednisone, dex: dexamethasone; FLS: fibroblast-like synoviocytes; CIA: collagen induced arthritis; cDNA: copy DNA; TNF: tumor necrosis factor; MMP: matrix metalloproteinase; P1NP: N-terminal propeptide of type 1 collagen; CTX1: collagen type *1* cross-linked C-telopeptide; PBMCs: peripheral blood mononuclear cells; IFN-α: interferon alpha; NF-ƙβ: Nuclear Factor kappa-light-chain-enhancer of activated B cells; mRNA: messenger ribonucleic acid; RA: rheumatoid arthritis; DAS28-4 (CRP): disease activity score using 28 joints and c-reactive protein with 4 variables; IL-6: interleukin 6; uNTX-1: N-terminal telopeptide 1 in urine; NA: not statistically analyzed; NS: not significant; S: significant; RCT: randomized controlled trial; ↑: results higher for SGRM compared to GC; ↓: results lower for SGRM compared to GC; =: results similar for SGRM compared to GC.

## Discussion

This paper aimed to systematically review the efficacy and safety of SGRMs compared to conventional GCs. We found 17 studies which investigated a SGRM compared to a GC in arthritis, of which seven showed similar efficacy and better safety compared to GCs. However only one SGRM, fosdagrocorat/PF-04171327, was investigated in a clinical setting.

There are several possible explanations as to why most of these SGRMs did not enter the clinical phase of drug development. One of them being the fact that some adverse effects associated with GC treatment are presumed to be caused by transrepression rather than by transactivation. For example, the immunosuppressive effects of GCs, leading to an increased risk of infections, are predominantly caused by transrepression rather than transactivation and therefore this clinically important adverse effect will not be reduced by a dissociative compound [36]. Other side effects, such as osteoporosis, are mediated by both transrepression (osteocalcin transcription) and transactivation (osteoblast apoptosis) [14]. Furthermore, transactivation is not only associated with negative effects, as it has been demonstrated that some genes that are upregulated by transactivation, such as mitogen-activated protein kinase phosphatase-1 (MKP-1, a crucial anti-inflammatory gene), GC-induced leucine zipper (GILZ, a protein which inhibits NFκB and AP-1) and the anti-inflammatory interleukine IL-10, have anti-inflammatory functions [37–39]. Thus, the actual effects of transrepression and transactivation are much more complex than suggested by the hypothesized working mechanism of SGRMs, in which it is claimed that transactivation is solely responsible for the adverse effects and transrepression for the desirable anti-inflammatory effects (full dissociation). Besides the described classical genomic mechanisms of action, which require several hours to take place, SGRMs also act by very rapid non-genomic mechanisms, especially at higher doses [40]. These non-genomic mechanisms of action are thought to be mediated by affecting the physicochemical property of cell membranes, or through binding to intracellular or membrane-bound GC receptor, causing inflammatory signal transduction cascades (mitogen-activated protein kinases (MAPK), neutrophil degranulation and phagocytosis by macrophages) [41–44]. Combined with epigenetic effects of SGRMs, these two mechanisms also contribute to the lack of dissociative effects of SGRMs. Furthermore, in vitro studies use simplified GRE reporter systems compared to the more complex GRE systems present in in vivo gene promoters [45]. Another important predicament in development of SGRMs is to establish equipotent doses of GCs and SGRMs. It has been shown that with increasing SGRM dosage, effects but also the SGRM-induced adverse effects increase [26,30] and vice versa. A case in point is deflazacort, an oxazoline derivative of prednisolone that was believed to have similar efficacy as prednisone but with fewer adverse effects, but in fact this actually proved to be at a lower than equipotent dosage; deflazacort even showed increased adverse effects compared to prednisolone in really equipotent dosages [46–47]. Furthermore, adverse effects measured in most of the experimental studies, such as increased glucose levels and changes in cortical bone thickness, are in fact surrogate markers for clinical adverse effects in patients, respectively development of diabetes mellitus and osteoporosis. Thus, these parameters in preclinical studies do not fully reflect the clinical GC-related adverse effects.

The only SGRM that did manage to enter a clinical phase in RA patients is PF-04171327 (NCT01393639), of which the first results of 12-week follow-up, were presented at the Annual European League Against Rheumatism (EULAR) Congress in 2015 [48]. In 323 RA patients, 15 mg of PF-04171327 daily showed similar efficacy as prednisone 10 mg daily, assessed by American College of Rheumatology (ACR) 20 response and Disease Activity Score 28 (DAS28), while (unwanted) effects on bone formation and plasma glucose level were similar as 5 mg of prednisone daily. These preliminary results suggest that development of SGRMs with a better efficacy/safety balance compared to GCs (better clinical anti-inflammatory efficacy and similar safety) is feasible.

The strengths of our study include the thorough search across several databases and inclusion of pre-clinical and clinical studies. The present systematic review is the first to investigate the benefit and risks of oral SGRMs compared to GCs in arthritis. A limitation of our review is the heterogeneity of the reported studies which made it difficult to compare these studies. We investigated efficacy (transrepression) by measuring effects of the SGRMs on inflammatory markers, and safety (transactivation) by measuring effects on glucose and bone metabolism. However, measurable effects on bone metabolism are more difficult to detect compared to effects on glucose levels in studies with short duration. This could be an explanation why only three of the 17 studies examined effect on bone markers.

In conclusion, studies assessing both efficacy and safety parameters of SGRMs in arthritis are scarce. There is limited evidence for dissociation of anti-inflammatory and metabolic effects of the SGRMs studied. Development of many SGRMs is haltered in a preclinical phase. One SGRM showed a better clinical efficacy/safety balance, compared to prednisone.

## Supporting information

S1 Box PubMed search for studies on efficacy and safety of selective glucocorticoid receptor modulators in comparison to glucocorticoids, in arthritis. (DOCX)

S1 Table Preferred Reporting Items for Systematic Reviews and Meta-Analyses (PRISMA) Checklist. (DOC)
