# Supplementary material for: Efficacy and safety of selective glucocorticoid receptor modulators in comparison to glucocorticoids in arthritis, a systematic review
Source: PLoS One. 2017 Dec 21;12(12):e0188810. doi: 10.1371/journal.pone.0188810 (PMC5739390; doi:10.1371/journal.pone.0188810)
Supplement: S1 Box — (DOCX) [file pone.0188810.s001.docx]

| **S1 Box 1. PubMed search for studies on efficacy and safety of selective glucocorticoid receptor modulators in comparison to glucocorticoids, in arthritis** |
| --- |
| (((((((((((((((((((((((((((((((((((((((selective glucocorticoid[Title/Abstract] OR "dissociated glucocorticoid agonists"[Title/Abstract]) OR "dissociated glucocorticoid receptor"[Title/Abstract]) OR "dissociated glucocorticoids"[Title/Abstract]) OR "selective glucocorticoids"[Title/Abstract]) OR "selective glucocorticosteroid"[Title/Abstract]) OR "sgrm"[Title/Abstract]) OR SGRMs[Title/Abstract]) OR SEGRM[Title/Abstract]) OR SEGRMs[Title/Abstract]) OR segra[Title/Abstract]) OR segras[Title/Abstract]) OR sgra[Title/Abstract]) OR sgras[Title/Abstract]) OR ("Receptors, Glucocorticoid/agonists"[Mesh] OR "Receptors, Glucocorticoid/antagonists and inhibitors"[Mesh])) OR "dagr"[Title/Abstract]) OR "dagrs"[Title/Abstract]) OR "selective gr agonist"[Title/Abstract]) OR "selective gr agonists"[Title/Abstract]) OR "selective gr antagonist"[Title/Abstract]) OR "selective gr antagonists"[Title/Abstract]) OR "selective gr ligand"[Title/Abstract]) OR "selective gr ligands"[Title/Abstract]) OR "selective gr modulator"[Title/Abstract]) OR "selective gr modulators"[Title/Abstract]) OR "non steroidal glucocorticoid receptor"[Title/Abstract]) OR "non steroidal agonist"[Title/Abstract]) OR "non steroidal agonists"[Title/Abstract]) OR "non steroidal gr antagonists"[Title/Abstract]) OR "non steroidal gr ligands"[Title/Abstract]) OR "non steroidal ligand"[Title/Abstract]) OR "glucocorticoid receptor/antagonist"[Title/Abstract]) OR "glucocorticoid receptor agonist"[Title/Abstract]) OR "glucocorticoid receptor ligand"[Title/Abstract]) OR "glucocorticoid receptor ligands"[Title/Abstract]) OR "glucocorticoid receptor modulator"[Title/Abstract]) OR "glucocorticoid receptor modulators"[Title/Abstract]) OR "dagra"[Title/Abstract]) OR "glucocorticoid receptor agonists"[Title/Abstract]) OR (segram[Title/Abstract] OR segrams[Title/Abstract])) AND ("arthritis"[MeSH Terms] OR Arthritis[Title/Abstract]) |
